# Influence of phenotypic variation of *Paenibacillus polymyxa* E681 on growth promotion in cucumbers

**DOI:** 10.3389/fmicb.2024.1427265

**Published:** 2024-07-31

**Authors:** Younmi Lee, Sungmoon Kwon, Kotnala Balaraju, Yongho Jeon

**Affiliations:** ^1^Department of Plant Medicals, Andong National University, Andong, Republic of Korea; ^2^Agricultural Science and Technology Research Institute, Andong National University, Andong, Republic of Korea

**Keywords:** *Paenibacillus*, phenotypic variation, temperature, growth promotion, root colonization

## Abstract

The goal of the current study is to better understand how bacteria may adapt to survive under adverse environmental conditions by altering and improving their phenotypes. In this study, we report the consequences of phenotypic variation in *Paenibacillus polymyxa* E681 (E681), a plant growth-promoting rhizobacterium (PGPR), isolated from winter barley root that has a variety of advantageous effects on crop plants. In our previous study, two different types of bacterial cells in E681 were distinguished. We used the term F-type for the variant that doesn’t produce endospores and B-type for the endospore-producing wild type. Under the circumstances of our experiment, the cucumber rhizosphere soil and the surface of the seeds produced phenotypic variance. On tryptic soy agar (TSA) plates, the B-type spontaneously converted into the F-type, but the reverse was not reversible. Intriguingly, the plant growth promotion test displayed that cucumber seedlings treated with F-type cells had characteristics resembling those of the untreated control. Whereas, growth promotion of cucumber seedlings treated with B-type depends on temperature conditions. In particular, an increased growth promotion was observed at a low temperature of 20°C. The phenotypic change from B-type to F-type did not occur at 20°C for 6 days in the growth curve analysis of E681, but it did occur on the fourth and second days at 30 and 37°C, respectively. Therefore, before using PGPR strains as a bacterial inoculant for sustainable agriculture, it is imperative to resolve phenotypic variance in these strains.

## Introduction

In numerous bacterial species, phenotypic variation allows for population diversity, which increases bacterial fitness under particular environmental conditions. It entails a high frequency of switches per cell every generation, which means that many cells in the population undergo swiching, leading to reversible ON/OFF transitions (Henderson et al., 1999). Furthermore, it is a natural adaptive process employed by several bacterial species to increase their population during changes in environmental conditions, which can result in the expression of several proteins (van der Woude and Baumler, 2004; van den Broek et al., 2005). Many bacteria with diverse ecological behaviors have been described as having phenotypic variation (Saunders et al., 2003; Li et al., 2012). Phenotypic variation has also been reported in Paenibacillus. *Paenibacillus lautus* NE3B01 has been observed to transition between cocci and motile rod forms, while *Paenibacillus vortex* exhibits variation into flagella-rich explorers and less motile builders (Roth et al., 2013; Mangwani et al., 2014).

However, the mechanisms underlying these variations remain poorly understood. Temperature is one of the numerous environmental stress factors that have a substantial impact on bacterial phenotypic variation. Bacteria adapt to different temperatures through complex cellular processes, altering their chemical phenotype, including membrane lipid composition, enzyme secretion, and pigment accumulation, as observed in ice- and snow-isolated strains (Smirnova et al., 2022). Furthermore, greater temperatures boost the metabolic rate of microorganisms (Nydahl et al., 2013). As a result, some bacteria may undergo genetic modifications that change their phenotypic and allow them to adapt to new environmental conditions. Soil microorganisms compete for nutrients and their biological niche on roots because they rely on organic compounds in the soil (Kuzyakov and Xu, 2013; Liu et al., 2016). Understanding the mechanisms of disease suppression and growth promotion is required before applying PGPR.

Plant growth-promoting rhizobacteria (PGPR) are root-associated microorganisms that have received a lot of attention. PGPR has favorable impacts on plants such as disease control and increased plant productivity (Kloepper et al., 2004; Hahm et al., 2012; Park et al., 2013). *Paenibacillus polymyxa* E681 was isolated from the roots of winter barley and identified as a PGPR strain capable of colonizing the rhizosphere of cucumber and *Arabidopsis* and exerting positive effects on these plants both *in vitro* and in field conditions (Ryu et al., 2003). *P. polymyxa* strains have been found to promote plant growth, manage plant diseases, and generate stress resistance when introduced into plants, such as *Arabidopsis* (Helbig, 2001; Ryu et al., 2005). Some *P. polymyxa* strains are thought to be vital for plant growth because they aid in the production of plant growth regulators including cytokinin and auxin (Lebuhn et al., 1997; Timmusk et al., 1999), as well as through colonizing the roots (Choi et al., 2004; Haggag and Timmusk, 2008). When bacteria colonize plant roots, they must be able to adapt to the complex and dynamic root environment (Sánchez-Contreras et al., 2002). Some bacteria, for example, may be able to produce a variety of enzymes that allow the breakdown of different forms of organic matter in the soil (Lladó et al., 2017). Other bacteria may be able to produce biofilms, allowing them to cling to root surfaces and protect themselves from environmental stress (Chao et al., 2022).

In our previous studies, E681 exhibited phenotypic variation during cultivation, with F-type generally appearing when growing B-type, which is the typical colony of E681. Interestingly, the change to F-type alters the original biological capacities of B-type, such as endospore and flagella formation, pH in culture, and carbon utilization.

Understanding the mechanisms and effects of bacterial phenotypic variation is critical for developing enhanced biotechnology solutions for plant disease management and plant development for sustainable agriculture. As a result, the goal of this work was to define phenotypic variation in *P. polymyxa* E681 at different temperatures and to assess the influence of phenotypic variation on root colonization and plant growth promotion in comparison to the wild-type.

## Materials and methods

### Microorganisms and culture conditions

Bacterium *Paenibacillus polymyxa* E681 (E681) was first isolated from winter barley roots in the southern part of Korea (Ryu and Park, 1997). When the B-type was cultured in TSB for 3 days at 28^°^C, F-type appeared. B-type and F-type were separately isolated and stored in a −80^°^C deep freezer for use in subsequent experiments.

### Effect of phenotypic variation of *P. polymyxa* E681 on root colonization and growth promotion under *in vitro* conditions

To test the root colonization capacity of phenotypic variation of *P. polymyxa* E681, two spontaneous rifampicin-resistant B- and F-types were grown on TSA media supplemented with rifampicin (100 μg/ml), and the plates were incubated at 28°C. Each B- and F-type can be clearly distinguished by their colony morphology on TSA and the presence or absence of endospore formation under a microscope. Colonies from the solid plates of each B-type and F-type were harvested and suspended in distilled water for use.. After surface sterilization of the seeds, B- and F-type were examined for their potential to colonize cucumber roots *in vitro* using seed coating and soil drench methods. Cucumber seeds were treated with 70% ethanol for 50 sec and washed twice with sterile distilled water (SDW). The seeds were then treated with 2% NaOCl for 10 min, followed by rinsing 3 times with SDW and air-dried on a clean bench. A B- and F-type bacterial culture grown on TSA for 48 h was collected in SDW and adjusted to a final concentration of 10^6^ cfu/ml. For the seed coating treatment method, the surface-sterilized cucumber (*Cucumis sativus* L. var. Eunsung-backdadagi) seeds were dipped in B- or F-type cell suspensions (10^6^ cfu/ml) or SDW (control) for 20 min under shaking conditions (180 rpm), and the seeds were then air-dried, transferred between two layers of wet papers, and incubated for two weeks. For the soil drench method, the surface-sterilized seeds were transferred to another set of falcon tubes containing the sterilized soil and inoculated with 5 ml of bacterial suspensions (10^6^ cfu/ml) in each falcon tube. SDW served as a non-treated control. The samples were incubated at 30°C under 8 h of light and 16 h of dark for 2 weeks to allow root development. For each treatment group, roots with soil removed were washed by shaking in distilled water for 30 seconds. Then, the 1 cm root tips were immersed in 0.1 M MgSO4 solution and ground using a mortar and pestle. After a 10-fold serial dilution, 100-μl aliquots were plated onto TSA plates and incubated at 30°C to determine colony-forming units (CFU). The number of bacteria colonizing the root was calculated as the cfu/g root tissue. One set of seedlings was used to measure the average fresh weight and length of seedlings in each treatment.

### Scanning electron microscopy analysis

The root tips of colonized cucumbers were taken for scanning electron microscopic (SEM) imaging two weeks following the treatment with B- and F-type bacterial suspensions using both seed coating and soil drench methods. Root tips were fixed in a fixative solution [50% EtOH, 5% (v/v) acetic acid, 3.7% (v/v) formaldehyde], washed twice in phosphate buffer (0.05 M, pH 7.2). The specimens were dehydrated using a series of ethanol gradients (20–100%) and coated with gold nanoparticles using an Ion coater. Thus prepared specimens were examined using a SEM (JSM-6400, Jeol Co., Japan) at 10, 15, and 20 kV.

### Assay of the plant growth-promoting capacity of B-and F-types under greenhouse conditions

The trial took place in a greenhouse near Andong National University, Korea. To investigate the effects of bacterial suspensions of B-and F-types on plant growth promotion in cucumbers grown in greenhouse conditions, cucumbers were grown using cultural methods such as drip irrigation and fertilizer as needed. There were three treatments with 3 replications per treatment and 20 plants per replication. The three treatments were B-type, F-type, and control. The experiment used transplants at two-leaf stage from plug-grown seedlings. Each type of bacterial suspension (106 cfu/ml) was drench on the soil three times with an interval of 2 weeks. Two weeks after the final treatment, the plant height and leaf number were measured. The experimental setup followed a completely randomized block design.

### Statistical analysis

Data were subjected to analysis of variance (ANOVA) using JMP software (SAS Institute Inc., Cary, NC, USA). The magnitude of the *F* value at *P* < 0.05 was used to determine the importance of B- and F-type treated plant growth factors. When a significant *F* value was achieved, the means were separated using Fisher’s protected least significant difference (LSD) at *P* < 0.05.

## Results

### Effect of phenotypic variation of *P. polymyxa* E681 on root colonization

**FIGURE 1:**
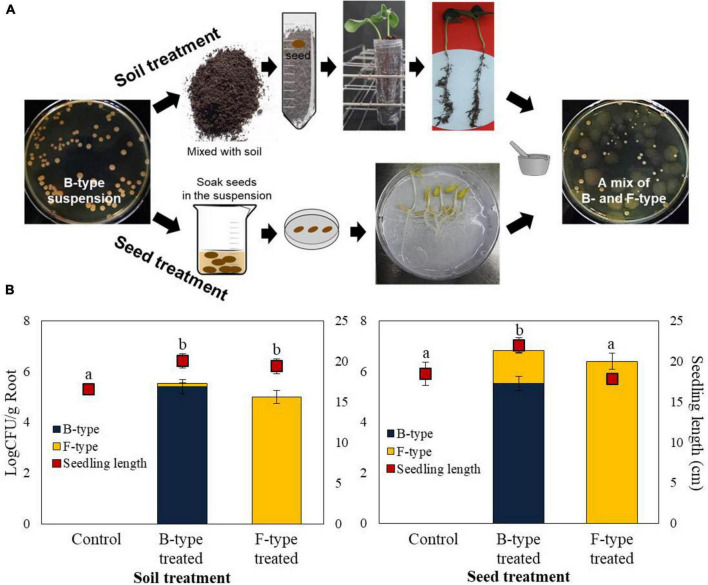
Effect of phenotypic variation on growth of cucumbers, and root colonization. **(A)** B- and F-types were tested for their ability to colonize cucumber roots *in vitro* by seed coating and soil drench methods after surface sterilization. **(B)** The number of bacteria colonizing the root was calculated as the cfu/g root tissue, and the average seedling length was measured. The seedling length was appeared to be greater in B-type-treated bacterial cells in comparison with F-type treatment. The experiment was performed at least two times with six replicates per treatment.

Under gnotobiotic conditions, root colonization was studied using two methods: seed coating and soil drenching (Figure 1A). Both treatments increased the amount of B- and F- type cells, while the control showed no root colonization. Regardless of whether the inoculationwas B-type or F-type, there were no statistically significant differences in the recovered population numbers. The total number of recovered cells from the B-type treatment was 3.5 × 10^5^ CFU/g root tissue, while that from the F-type treatment was 1.0 × 10^5^ CFU/g root tissue using the soil drench method (Figure 1B). Through seed coating treatment, the B-type cells colonized more efficiently (6.4 × 10^6^ CFU/g root tissue) than the F-type cells (2.5 × 10^6^ CFU/g root tissue) (Figure 1B). Phenotypic variation occurred in both treatment methods. In all procedures, mixed colonies of B- and F-type developed from the roots of seedlings treated with B-type cells. This finding indicated that the phenotypic variation can occur not only on artificial medium but also on seed and soil treatments. The number of F-type cells recovered after B-type cells treatment in the soil drench method was 8.5 × 10^3^ CFU/g, accounting for 2.4% of the total. The number of F-type cells recovered following B-type treatment in seed coating was 1.7 × 10^6^ CFU/g, accounting for 26.0% of the total.

### Scanning electron microscopy (SEM)

**FIGURE 2:**
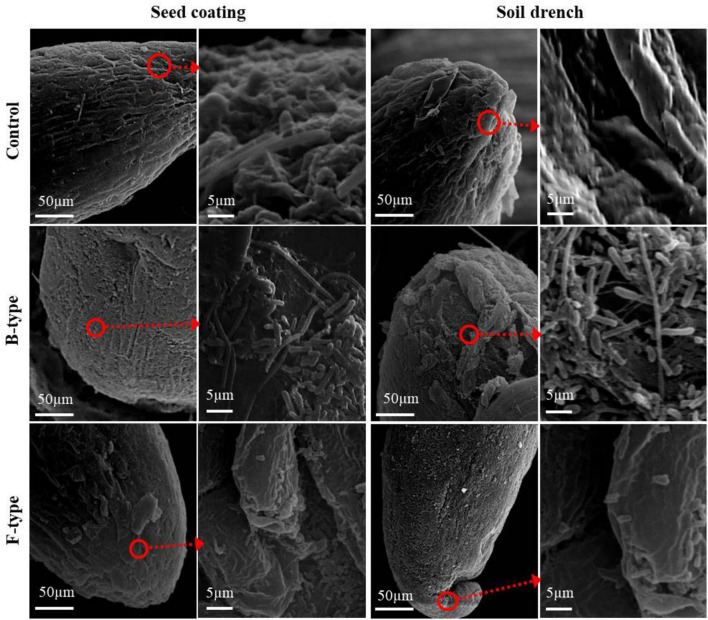
Microscopy analysis. Scanning electron microscopy (SEM) of cucumber root tips colonized by B- and F-type bacterial cells two weeks after treating the seeds by the seed coating and soil drench methods. Root tips were colonized by the bacterial population at a higher level for B-type compared with the F-type cells, while no bacterial population was observed in the non-treated control. Dotted lines indicate enlarged photos. The experiment was performed at least two times with six replicates per treatment.

SEM of bacterial associations colonizing root tissues revealed that the root tips were colonized by rod-shaped bacterial cells classified as E681 in comparison to the non-treated control in which the roots were not colonized. However, in both treatments, the roots were colonized with a greater number of B-type cells than F-type cells (Figure 2).

### Effect of phenotypic variation of *P. polymyxa* E681 on growth promotion of cucumbers

**FIGURE 3:**
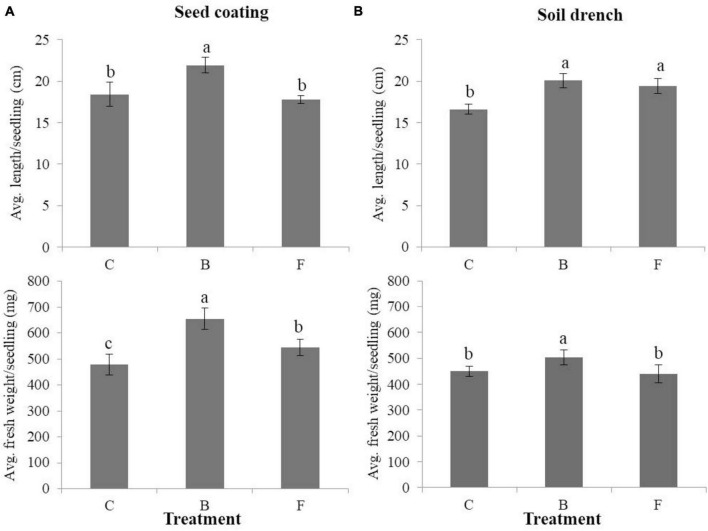
Effects of phenotypic variation on plant growth promotion under *in vitro* conditions in cucumbers. The average length and fresh weight/seedling in B- and F- treated cucumbers using the seed coating **(A)** and soil drench **(B)** methods with suspensions in comparison with the non-treated control. Each treatment contains three replicates, and the experiment was performed at least twice. Bars with the same letters are not statistically different from each other, according to the least significant difference test (*P* < 0.05).

**FIGURE 4:**
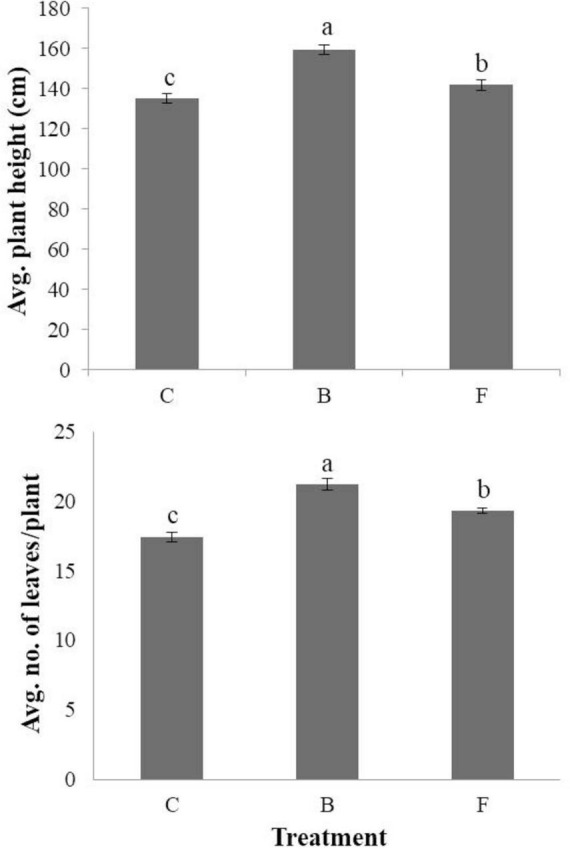
Effects of phenotypic variation on plant growth promotion under greenhouse conditions in cucumbers. The average stem length and the average number of leaves per plant under greenhouse condition in B- and F-treated plants in comparison with the non-treated control. Each experiment comprised three replicates, and the experiment was performed at least twice. Bars with the same letters are not significantly different from each other, according to the least significant difference test (*P* < 0.05).

The average seedling length (cm) and fresh weight (mg) per seedling were measured for both B- and F-treated seedlings, as well as non-treated control plants, using seed coating and soil drench methods in cucumbers (Figure 3). When the seed coating method was applied, the B-type treatment outperformed the F-type treatment and the untreated control in terms of average length and fresh weight per seedling. Seedlings with the B-type treatment had a length of 21.9 cm and a fresh weight of 655.6 mg, whereas those with the F-type treatment had a length of 17.8 cm and a fresh weight of 543.8 mg. The soil drench method, on the other hand, produced seedlings with larger average lengths than the untreated control (Figure 3B) for both B- and F-type treatments. In particular, for the non-treated control, B- and F-type treatments, the average seedling lengths were measured as 16.6, 20, and 19.4 cm, respectively, and the average fresh weights were measured as 449.6, 503.1, and 439 mg, respectively. The study evaluated how B- and F-type bacterial suspensions affected the stimulation of plant growth in cucumbers cultivated under greenhouse conditions. Using a soil drench method, the plant height and leaf count were measured for both B- and F-type treatments. In comparison to the F-type treatment, the results showed that the B-type treatment significantly increased plant height and number of leaves per plant (Figure 4). Therefore, it was discovered that the B-type treatment was superior to the F-type as a plant growth-promoting rhizobacteria (PGPR) in both *in vitro* and greenhouse conditions.

## Discussion

In general, microbes can increase their population-level diversity to assist themselves survive in adverse environmental conditions. A number of properties, including shape, growth rate, motility, antibiotic resistance and pathogenicity can be influenced by bacterial phenotypic differences. For instance, changes in gene expression patterns can have an impact on the production of enzymes or other proteins, whereas bacterial mutations can modify the size, shape, or color of cells (Hughes and Andersson, 2017). This phenotypic diversity is crucial for niche adaptation and is required to increase intra-population diversity, which raises bacterial fitness. Endospores serve as a primary survival strategy for microbes to endure extreme conditions such as high temperatures, low temperatures, desiccation, and high salinity (Nicholson et al., 2000). They enable microbes to persist in harsh environments and are crucial in the development of biological control agents for enhancing stability and efficacy in the field. In previous studies, it was observed that the B-type of E681 forms endospores, whereas the variant F-type does not form endospores at all. We hypothesized that E681 bacteria may adopt a survival strategy by favoring increased flagella formation and mobility under unfavorable conditions instead of committing to sporulation. This adaptation could potentially reduce the necessity for biofilm formation or antibiotic production. In comparison to the B-type, the F-type bacteria showed decreased abilities in biofilm formation and antibiotic production. This enhanced motility is attributed to the lack of expression of sporulation-related genes at stage 0 of sporulation, which conserves energy that can then be redirected towards increasing flagella production and other metabolic activities. Further extensive research is required to substantiate this hypothesis. In the case of E681, following several subcultures on the TSA medium, the bald and convex-shaped colony (B-type) switched to the flat-shaped colony phenotype variant (F-type). According to Li et al. (2012), during the phenotypic variation, the variants are typically unstable and possible to develop into the original phenotype, but the switch can occasionally be irreversible (Wisniewski-Dye and Vial, 2008). In our previous study, the phenotypic variation phenomenon in E681 does not occur through genetic mutations. Since reversion from F-type to B-type does not occur, it is likely a phenomenon of epigenetic variation. In-depth research into methylation or adenylation seems necessary. Similar to this, even after numerous subcultures, we found no reversion of the phenotypic variant (F-type) to wild-type (B-type) in our investigation. Due to its significant biotechnological potential in various industrial processes and in sustainable agriculture, *P. polymyxa* is a well-studied PGPR that has attracted considerable interest (Lal and Tabacchioni, 2009). However, from the perspective of biological control agents developers, the phenotypic variation from B-type to F-type is viewed unfavorably by developers of Biological Control Agents (BCAs) due to the fact that F-type does not form endospores, which can negatively impact the stability of these biological control agents. The variance in bacterial phenotypes can also be influenced by environmental factors like temperature or the availability of nutrients. For instance, certain bacteria have the ability to produce spores that allow them to survive in harsh environments such extremely low nutrition levels or high temperatures (Lee et al., 2020).

Every day, the number of F-type colonies that developed after serial dilutions and plating of the B-type cultures on TSA plates was tallied. F-type occurred after two days and 4 days, respectively, of culture at 37 and 30°C, while it took 6 days of observation at 20°C to see F-type variation. Once phenotypic variation begins, nearly 100% of B-type cells have switched to F-type. We confirmed that there was no colony variation after culturing B-type on solid medium at 28^°^C for 2 days, and used it for PGP experiments. The plant growth temperature was 25^°^C, which we believe to be the most suitable condition for both plant and E681 growth. According to Huo et al. (2010), temperature conditions were among the environmental factors that caused *P. polymyxa* SQR-21 to sporulate. Each phenotypic variant has unique optimal temperatures and medium requirements for survival, according to a prior study by Berditsch et al. (2007). This is particularly true for *Bacillus brevis*, whose optimal growth temperature ranges from 40 to 42°C. As a result, temperature has a significant impact on how new phenotypes are formed. According to a study by Alvarez et al. (2006), two more *Paenibacillus* species, *P. peoriae*, and *P. polymyxa*, displayed growth variations based on temperature, pH, and inoculum density level. Additionally, it has been shown that colony morphology conversion affects growth and varies among phenotypic variants. For instance, *Streptococcus* GAS734 and GAS968, which both exhibit phenotypic instability, were found to have distinct growth curves (Chiang-Ni et al., 2014).

Many PGPR strains have been reported to exhibit phenotypic diversity, which is a widely prevalent phenomenon that influences root colonization (Lerner et al., 2010; Volfson et al., 2013). Successful root colonization is a prerequisite for PGPR to have an advantageous impact on plant growth (Rothballer et al., 2008). As evidenced by the dominant root colonization of cucumber roots compared with the F-type in both the seed coating and soil drench treatments, competitive root-colonization behavior was found with the B-type at a greater degree. Under gnotobiotic circumstances, the B-type was superior to the F-type for promoting cucumber growth in terms of average length and fresh weight per seedling. This finding was further confirmed by the ability of the B-type to produce higher levels of IAA than the F-type (Lee et al., 2020). Similarly, Huo et al. (2010) found an increase in wheat crop output when IAA-producing rhizobacteria were administered to the plant roots. Nevertheless, B-type did not promote plant growth by the seed coating method in greenhouse conditions. It implied that F-type, which has poor plant growth promotion happened, and that temperature was one of the factors impacting the phenotypic variation occurrence. Highly competitive colonization of plant roots via bacterial cell invasion is critical for effective bacteria-plant interactions (Kamilova et al., 2005). According to a recent study by Kefela et al. (2015), *A. thaliana* plants treated with *P. polymyxa* grew substantially larger than non-treated controls. Bacteria with advantageous phenotypic features can promote plant growth by producing plant growth-promoting compounds, nitrogen fixation, nutrient solubilization, and pathogen suppression (Costa et al., 2014; Souza et al., 2015). Bacteria with detrimental phenotypic features, on the other hand, might have a negative impact on plant growth promotion by producing toxins or plant growth inhibitors, competing for nutrients with plants, or increasing the growth of plant diseases (Bittencourt et al., 2023). As a result, the influence of phenotypic variation on plant growth promotion is determined by the unique characteristics of the bacteria involved. To optimize the advantages and limit the potential negative effects, it is critical to choose and use bacteria with desirable phenotypic features for plant growth promotion.

In conclusion, our results indicated that B-type could change into a phenotypic variant F-type on the surface of seeds or in the soil. Even after several subcultures, there was no reversion of the F-type to the B-type. Furthermore, the B-type outperformed the F-type in terms of root colonization and promoting plant growth. Prior to being commercially made available for sustainable agriculture, beneficial microorganisms like *P. polymyxa* E681 must be thoroughly investigated to better understand the traits of their variants.

## Data availability statement

The raw data supporting the conclusions of this article will be made available by the authors, without undue reservation.

## Author contributions

YL: Conceptualization, Investigation, Methodology, Writing – original draft. SK: Investigation, Methodology, Writing – original draft. KB: Data curation, Validation, Writing – review & editing. YJ: Funding acquisition, Project administration, Supervision, Writing – review & editing.
